# A Hybrid Pragmatic and Factorial Cluster Randomized Controlled Trial for an Anti-racist, Multilevel Intervention to Improve Mental Health Equity in High Schools

**DOI:** 10.1007/s11121-023-01626-x

**Published:** 2024-01-04

**Authors:** Marta I. Mulawa, Sharron L. Docherty, Donald E. Bailey, Rosa M. Gonzalez-Guarda, Isaac M. Lipkus, Schenita D. Randolph, Qing Yang, Wei Pan

**Affiliations:** 1 Duke University School of Nursing, Duke University, Durham, NC USA; 2 Duke Global Health Institute, Duke University, Durham, NC USA; 3 Department of Pediatrics, Duke University School of Medicine, Durham, NC USA; 4 Department of Population Health Sciences, Duke University School of Medicine, Durham, NC USA

**Keywords:** Multilevel interventions, Mental health equity, Systemic racism, Youth, Cluster randomized controlled trial

## Abstract

Systemic racism is pervasive in US society and disproportionately limits opportunities for education, work, and health for historically marginalized and minoritized racial and ethnic groups, making it an urgent issue of social justice. Because systemic racism is a social determinant of health prevalent across multiple social and institutional structures, it requires multilevel intervention approaches using effective designs and analytic methods to measure and evaluate outcomes. Racism is a fundamental cause of poor health outcomes, including mental health outcomes; thus, mental health services and programs that address racism and discrimination are key to promoting positive mental health of racial and ethnic minority youth. While multilevel interventions are well-suited for improving outcomes like youth mental health disparities, their evaluation poses unique methodological challenges, requiring specialized design and analytic approaches. There has been limited methodological guidance provided to researchers on how to test multilevel interventions using approaches that balance methodological rigor, practicality, and acceptability across stakeholder groups, especially within communities most affected by systemic racism. This paper addresses this gap by providing an example of how to rigorously evaluate a hypothetical, theoretically based, multilevel intervention promoting mental health equity in three US school systems using an anti-racist approach intervening at the macro- (i.e., school system), meso- (i.e., school), and micro- (i.e., family and student) levels to improve mental health in adolescents. We describe the design, sample size considerations, and analytic methods to comprehensively evaluate its effectiveness while exploring the extent to which the components interact synergistically to improve outcomes. The methodological approach proposed can be adapted to other multilevel interventions that include strategies addressing macro-, meso-, and micro-levels of influence.

**Supplementary Information:**

The online version contains supplementary material available at 10.1007/s11121-023-01626-x.

## Introduction

Systemic racism is pervasive in US society and continues to disproportionately limit opportunities for education, work, and health for historically marginalized and minoritized racial and ethnic groups, making it an urgent issue of social justice (Wright et al., 2020). Inclusive of internalized, interpersonal, and institutional racism, systemic racism is the impact of formal and informal policies and procedures that facilitate and empower organizations to engage in discriminatory acts (Bailey et al., 2021). Scholars agree that it should be the focus of interventions aiming to curb racism and its effects on health inequities (Brown et al., 2019). Because systemic racism is prevalent across multiple social and institutional structures, it requires multilevel intervention approaches using effective designs and analytic methods to measure and evaluate outcomes (Adkins-Jackson & Incollingo Rodriguez, 2022). Multilevel interventions can target risk and protective factors for health at multiple levels and address the historical and current environmental context in which individuals work, live, thrive, or play, commonly referred to as the social determinants of health (SDoH) (Johnson-Jennings et al., 2023). Approaches curtailing systemic racism must disentangle at the system level, policies, and practices embedded in the existing societal and organizational structure, favorable to a dominant group and unfavorable to other groups, while concurrently, or sequentially addressing determinants and bolstering resilience within lower levels of influence (e.g., schools and families).

Recent studies have shown racism is a fundamental cause of poor health outcomes, including mental health outcomes (Williams et al., 2019). Additionally, Black students are more likely to be disciplined and expelled in school and placed in juvenile detention facilities than White students (Aronowitz et al., 2021). For Black males, media portrayals as a predator and low expectations in formative environments (e.g., schools) may become internalized as personal narrative. As such, experiences of discrimination and marginalization become normalized and manifest as risky behaviors. Racism and racial disparities have also been linked to the development of affective, psychotic, and substance use disorders (Duncan et al., 2023; Godbolt et al., 2022; Paradies et al., 2015). Black and Hispanic/Latino children experience disparities in mental health service utilization, having 1.5–3 times the odds of having an unmet mental health need than White children (American Psychological Association, 2018; Kataoka et al., 2002). Mental health services and programs that address racism and discrimination are key to promoting the positive mental health of racial and ethnic minority youth. An explicit anti-racist approach that recognizes and addresses racist structures influencing mental health is sorely needed. Community-based settings such as high schools are an environment in which factors at macro- (e.g., school system), meso- (e.g., school), and micro- (e.g., family and student) levels combine synergistically to negatively influence mental health. High schools are critical contexts to influence racism to improve mental health using approaches that build trust and foster collaboration between mental health providers and communities (Hansen et al., 2018, p. 201). Indeed, there is emerging evidence identifying community and parental anti-racism as impactful protective factors (Heberle et al., 2022) for youth mental health.

While multilevel interventions are well-suited for improving outcomes like youth mental health disparities, their evaluation poses unique methodological challenges and opportunities, requiring specialized design and analytic approaches (Dye et al., 2019). In the case of a multilevel intervention implemented in high schools, all students within the same school are likely to be impacted by an intervention delivered at the school-level (e.g., school specific policy change). As a result, to evaluate the effects of an intervention delivered at the school level, students within the same school must be allocated as a group, or cluster, to receive the school-level intervention or a school-level comparison condition. Cluster randomized controlled trials (cRCTs) are the gold standard for evaluating multilevel interventions that require the allocation of groups to a treatment condition (Murray et al., 2004). Designing a cRCT that is sufficiently powered to test intervention effects must account for the extent to which outcomes of individuals within a cluster are similar to each other, known as the intraclass correlation coefficient (ICC) (Turner et al., 2017a, b). Similarly, evaluating data collected through a cRCT must employ analytic approaches that account for nesting of individuals within clusters (Turner et al., 2017a, b).

Other interventions, particularly those delivered at the macro- (i.e., system/policy) level, may not be optimal candidates for evaluation through a cRCT because many clusters are simultaneously exposed to the intervention and financial/logistical constrains limit the possibility of engaging and randomizing a sufficient number of macro units. In such circumstances, one feasible approach is to implement a pragmatic pre-post design for evaluation purposes at the macro-level, followed by a cRCT evaluating other intervention components at the meso- and micro-levels.

Additionally, randomizing students within a school to receive a micro- (i.e., family) level intervention or control condition poses its own challenges in which the intervention is inadvertently received by students who were randomized to the control condition, resulting in contamination. Such contamination can shift outcomes in the control group in the same direction as those in the intervention group, leading researchers to under-estimate the effects of an effective intervention. In such circumstances, care must be taken to minimize and measure possible contamination.

Another consideration in the evaluation of multilevel interventions is that the interventions often involve complex and multifaceted interacting components. These components, typically operating at various levels, are often hypothesized to interact to influence health outcomes. Indeed, understanding the extent to which interventions impede or enhance each other is often of great interest to potential program implementers; such findings have implications for maximizing constrained resources. However, most multilevel interventions are evaluated as a package, with trials unable to estimate independent or joint effect of intervention components (Agurs-Collins et al., 2019). Estimating such effects may be accomplished with a factorial cRCT design, a powerful yet underutilized experimental design that allows for the evaluation of more than one intervention component in a single study (Mdege et al., 2014). While factorial cRCT designs have limitations, including requiring a larger sample size than needed in a parallel design comparing a full intervention to a control, they do have notable strengths, including an important advantage from an equity perspective: that a larger proportion of clusters (e.g., 75% in a 2 × 2 factorial design) are randomized to receive at least one intervention component compared to the 50% that would be randomized to receive the full multilevel intervention using a standard parallel cRCT design (Crespi, 2016). This characteristic may make a factorial cRCT design more desirable and acceptable to community members, which may lead to greater participation.

This paper describes design and analytic methods to evaluate the effectiveness of a hypothetical multilevel intervention, intervening at the macro- (i.e., system), meso- (i.e., school), and micro- (i.e., family and student) levels to improve mental health in adolescents. Rigorously evaluating a complex multilevel intervention implemented in community-based setting, while exploring the extent to which the components interact synergistically to improve outcomes, requires balancing design rigor with pragmatics of conducting a study that is sensitive to community-identified needs. We describe an approach that feasibly and comprehensively achieves this objective.

## Methods

### Intervention

**Fig. 1:**
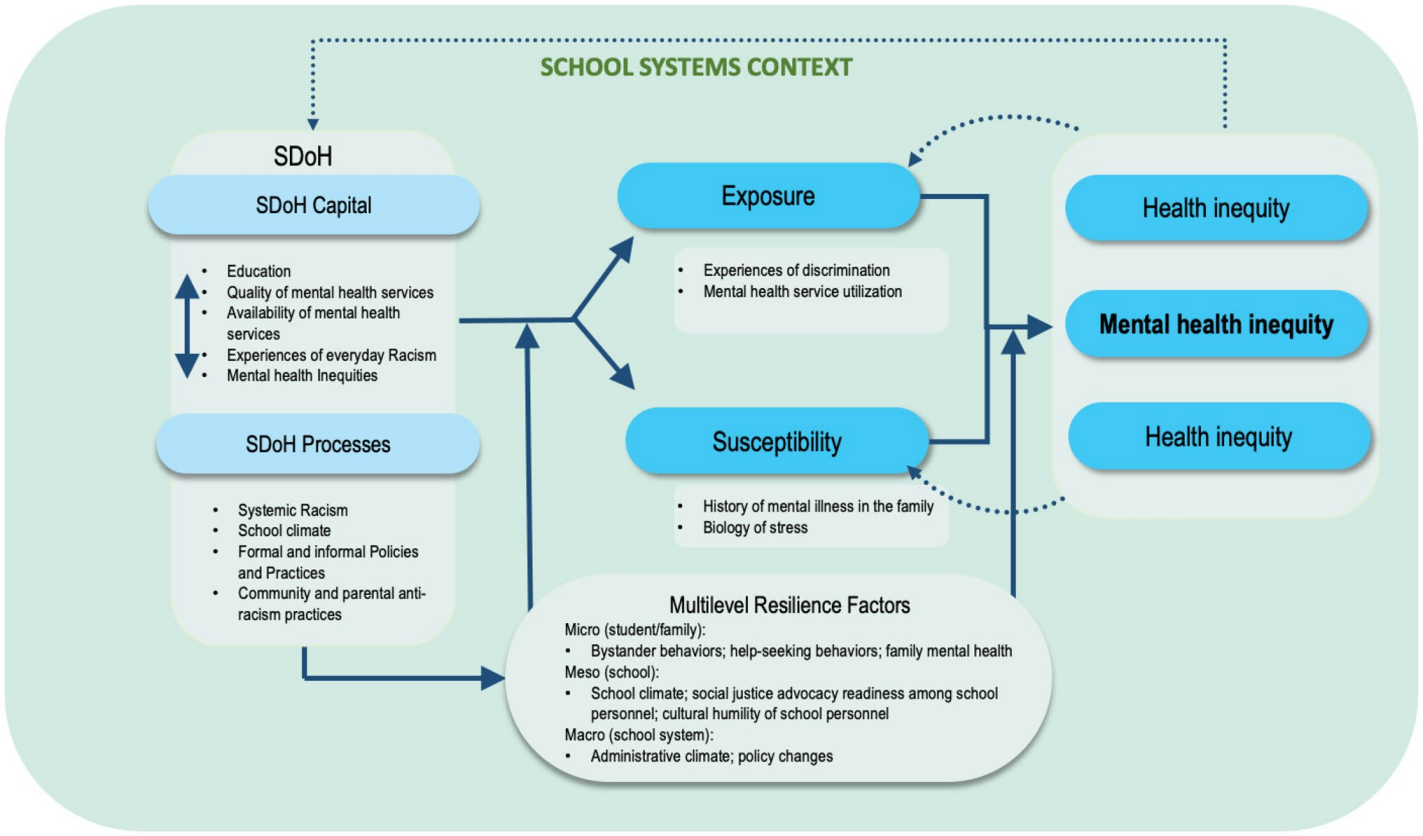
Adapted framework for operationalizing Social Determinants of Health (SDoH) mechanisms and multilevel resilience factors to reduce mental health inequity

The hypothetical intervention is a community-based, multilevel intervention informed by an adapted heuristic framework for conceptualizing and operationalizing SDoH mechanisms to inform the development of interventions that mitigate harmful SDoH and support strength-based resilience factors to reduce health inequities (Guilamo-Ramos et al., 2023; Thimm-Kaiser et al., 2023). Our adaptation of this framework (Fig. 1) acknowledges the importance of *SDoH capital*, socially distributed resources that affect mental health inequities, like education and the quality and availability of mental health services. The framework also informs our focus on addressing *SDoH processes*, conceptualized as the factors that shape interactions between people that influence those outcomes, partly by influencing access to capital. In our case, *SDoH processes* include systemic racism, school climate, formal and informal policies and practices within schools, and community and parental anti-racism practices. The framework illustrates how these SDoH capital and processes impact *exposure*, conceptualized as contact that causes a risk to one’s health or serves to protect health outcomes (e.g., experiences of discrimination and mental health serve utilization) and *susceptibility*, considered to be biological factors that influence the likelihood of morbidity (e.g., family history of mental illness). Most importantly, and consistent with the framework’s intention (Thimm-Kaiser et al., 2023), our intervention includes strategies that promote multilevel resilience factors operationalized across macro-, meso-, and micro-levels of the social ecology of high schools, thus having greater potential to achieve positive outcomes than a single level intervention alone (Komro et al., 2016).

The hypothetical intervention will be evaluated in three public school systems, including the Durham County Public School System in Durham, North Carolina. Researchers will work in close collaboration with school system leaders supporting diversity, equity, and inclusion (e.g., the Durham Public School’s Office of Equity Affairs). The Durham County Public School System comprised 12 high schools with a diverse racial and ethnic composition of 42% Black/African American, 33% Hispanic/Latino, 19% White, and 6% other racial and ethnic groups. Two additional school systems will be purposefully selected to be approximately similar in size, resulting in approximately 30 high schools with comparable racial and ethnic compositions.

Three integrated intervention components will be delivered across three levels (macro, meso, micro) with a focus on the contextual effects of policies and practices at the macro- (i.e., system) level. At the macro-level, we will implement restorative justice talking circles, an evidence-based strategy that increases awareness of consequences of oppressive educational structures and disciplinary actions on students and families, promotes anti-racist leadership, and leads to policy change (Lowe et al., 2022; Mansfield et al., 2018; Marcucci, 2021). The talking circles will include school district and school administrators from all 30 high schools. In these talking circles, administrators will be guided by an expert consultant to examine school policies and procedures that facilitate discriminatory acts and inequity across race and ethnic groups using a Racial Equity Impact Assessment (REIA) (Racial Equity Impact Assessment Toolkit, 2009). A REIA is a systematic evaluation of how a proposed action or decision may impact different racial and ethnic groups. The goal of using a REIA is to minimize negative, unanticipated consequences from proposed policies, institutional practices, programs, plans, and financial decisions. The REIA can also be a tool for preventing institutional racism and for identifying opportunities to address inequities.

At the meso- (i.e., school) level, we will implement training in racial justice, cultural humility, and mental health first aid for school administrators, faculty, and staff. The purpose of this training is to increase inclusive practices among school personnel, which is expected to decrease experiences of discrimination and improve mental health outcomes among students (De Jesús et al., 2016; Pham et al., 2022). This training will ensure that school administrators and staff have the knowledge and skills needed to prevent racial biases from affecting the educational quality and mental health services, as well as examine how policies and procedures are implemented in the school system. The training will cover self-awareness regarding implicit biases, review and discussion of school policies and procedures, and skills related to recognizing signs of mental health challenges and partnership-building with families and students.

At the micro- (i.e., family) level, our intervention will include mental health first aid training for families. Families can be a source of resilience and support for youth and are uniquely positioned to buffer negative impacts of systemic racism and other stressors on mental health outcomes among youth (Healy et al., 2018). This family-based intervention will teach families how to identify and understand signs of mental health challenges and equip them with skills to respond to such challenges by providing support and advocating for students experiencing challenges. The intervention will improve mental health by increasing psychological well-being and hopefulness while improving coping skills and help-seeking behaviors from trusted adults (Morgan et al., 2018).

### Design, Measures, and Analytic Sample

We will employ a multiphase, hybrid pragmatic and factorial cRCT to evaluate the effectiveness of the anti-racist, multilevel mental health intervention (Table 1). Our proposed design will employ a 1-year pretest–posttest pragmatic trial to evaluate an intervention for school system’s policy change at the macro-level in year 1, followed by another 1-year 2 × 2 cross-level factorial cRCT to evaluate the effects of intervention strategies delivered at the meso- and micro-levels focusing on the primary outcome, adolescent mental health, in year 2. Both phases will be conducted with 30 clusters (i.e., high schools in three school systems).

**Table 1:** Overview of multilevel intervention and study design

Phase	Level	Socio-cultural context	Intervention	Design	Intervention participants	Outcome	
						**Concept**	**Measure**
I	Macro	Public school system	Talking circles and REIA	Pretest–posttest pragmatic design	School board members School administrators School personnel	*Administrative climate Policy changes	Surveys Interviews with faculty, students, staff
						Adolescent mental health	Children’s Depression Inventory (CDI) Reynolds Children’s Manifest Anxiety Scale (RCMAS)
II	Meso	High schools	Training in racial justice, cultural humility, mental health first aid for school personnel	One-year pretest–posttest factorial cRCT for multilevel intervention. cRCT at meso-level for intervention vs control Stratified RCT at micro level within each school for intervention vs. control	School administrators Faculty Staff	Racial equity, knowledge, attitudes, beliefs, self-efficacy	Multifactor Racial Inventory
						Cultural humility	Cultural Humility Scale
						Social justice advocacy readiness	Social justice Advocacy readiness questionnaire
						School climate	Interviews with faculty, students, staff
						*Adolescent mental health	Children’s Depression Inventory (CDI) Reynolds Children’s Manifest Anxiety Scale (RCMAS)
	Micro	Home	Mental health first aid		Families Students	Family mental health	The Family Health Scale
						Bystander behaviors Self efficacy	Situational Attitudes Scale
						Adolescent psychological wellbeing Connectedness Hopefulness	Ryff Psychological Wellbeing Scale Engagement, Perseverance, Optimism, Connectedness and Happiness Measure
						*Adolescent mental health	Children’s Depression Inventory (CDI) Reynolds Children’s Manifest Anxiety Scale (RCMAS)

^*^The primary outcome for the corresponding intervention level

In phase I, all 30 schools will receive a 1-year macro-level intervention centered around restorative talking circles and REIA to improve school policy and climate for better youth mental health outcomes. The analytic random sample for phase I will include (1) 30 school system administrators (ten from each of the three school systems), (2) 300 high school personnel (i.e., school administrators, teachers, and/or staff; ten from each of the 30 schools), and (3) 450 ninth grade students and families (15 from each of the 30 schools). We focus on 9th grade students because the intervention is likely to have a larger impact during a time when students are acclimating to their new school environments (Bailey et al., 2020). Participants in phase I will complete a pretest assessment at the start of the school year, prior to the implementation of intervention activities, as well as a posttest assessment at the end of the school year. A subsample of phase I participants will be recruited for participation in qualitative interviews, including all 30 school system administrators and a purposive stratified sample of 60 teachers, 60 students, and 30 school personnel, a qualitative sample size sufficient for theme saturation (Small, 2009). We will stratify the sample to ensure variability in factors that may influence experiences with the macro-level intervention (e.g., race, age, time at their school).

In phase II, all clusters (i.e., schools) will be equally randomized to receive the meso-level intervention or control. For this phase, we will recruit a new analytic random sample comprised of *N* = 3060 families of tenth grade students served through the school systems (i.e., a new random sample of *n* = 102 tenth grade students/families within each of the *k* = 30 schools). Within each of these schools, the selected families will be randomized to either the micro-level intervention or control. To maximize the intervention’s effects, the intervention will be delivered to the entire family unit, though data will be collected from one parent or legal guardian. We will also recruit high school personnel (i.e., school administrators, teachers, and staff (*n* = 450 within *k* = 30 schools) to measure other secondary outcomes of interest (e.g., cultural humility, social justice advocacy readiness). Participants in phase II will complete assessment at the start of the school year (the pretest assessment) and at the end of the school year (the posttest assessment). Detailed constructs of interest and corresponding measures are described in Table 1. This design will allow us to rigorously test the main effects of each level of intervention and explore their interaction. For example, examining the cross-level interaction effect between a micro-level intervention (e.g., mental health first aid training for families) and a meso-level intervention (e.g., training in racial justice, cultural humility, and mental health first aid for school administrators, teachers, and staff) will help us understand whether students who are in a school that received the meso-level intervention as well a family that receive the micro-level intervention will report greater mental health improvements from the multilevel intervention than students who only received one level of the intervention. In phase II, the estimated policy changes from phase I will be also considered as a key factor. This link between the two phases will allow us to examine the impact of the effect of the macro-level intervention evaluated in phase I on the effect of the subsequent meso- and micro-level interventions evaluated in phase II. For both phases, if dropouts exist from the pretest assessment to the posttest assessment, oversampling participants can be adopted to accommodate the dropouts, or missing data imputation techniques can be applied for the posttest assessment if oversampling is not feasible.

### Analyses

As shown in Table 2, we will conduct three-level multilevel modeling (MLM) thrice to analyze the data from phase I’s 1-year pre-post design for assessing the effect of the pragmatic intervention for all 30 schools in the three school systems on school system’s policy change perceived by students, families, and school personnel. For example, a three-level MLM on students’ perceptions of school system’s policy changes can be specified as follows (see S1 in Supplementary Information for three-level MLMs on families’ and school personnel’s perceptions of school system’s policy changes):

1 $${Y}_{tij}=\mu +{\alpha }_{j}+\theta \times {{\text{Time}}}_{tj}+\sum_{p=1}^{P}{\gamma }^{(p)}\times {S}_{j}^{(p)}+\sum_{q=1}^{Q}{\varphi }^{(q)}\times {C}_{ij}^{(q)}+{\varepsilon }_{tij}$$

where

**Table 2:** Overview of analytic methods

Phase	Level/unit of analysis	Schema of random sampling	Key factor	Main outcome	Covariates	Analytic method
I	1 (Time)	Pretest–posttest measures	Time	Policy change perceived by • Students • Families • School personnel • School system administrators		• 3-level MLM (*for student, family, or school personnel outcomes separately*) • Qualitative analysis (*for* school system administrators)
	2 (Individuals: *students/families, school personnel, or school system administrators*)	• 450 students/families (*15 from each of 30 schools*) • 300 school personnel (*10 from each of 30 schools*) • 30 school system administrators (*10 from each of the 3 school systems*)			Individual characteristics	
	3 (Schools)	All 30 schools receiving intervention			School characteristics including the indicator of the 3 school systems	
II	1 (Individuals: *students/families or school personnel*)	• 1530 students/families (*51 from each of the 30 schools*) receiving Micro intervention + 1530 students/families (*51 from each of the 30 schools*) receiving no intervention • 450 school personnel (*15 from each of the 30 schools*)	• MESO • MICRO • MESO × MICRO	Posttest assessment of • Adolescent mental health • Family mental health • School personnel cultural humility, social justice and advocacy, and school climate	Individual characteristics including perceived school system’s policy change estimated from Phase I (MACRO)	• 2-level MLM (*for student, family, or school personnel outcomes separately*) with controlling for pretest assessment at Level 1
	2 (Schools)	15 schools receiving Meso intervention + 15 schools receiving no intervention			School characteristics including the indicator of the 3 school systems	

*Y*_*tij*_ is the outcome (perceived school system’s policy change) for the *i*th student at time *t* in the *j*th school (*i* = 1, …, *n*_*i*_; *j* = 1, …, 30, *t* = 0, 1) and for this phase, *n*_*j*_ = 15 students;

*μ* is the mean of *Y*_*tij*_ in the pre-intervention condition at time 0;

*α*_*j*_ is a random intercept for the *j*th school such that *α*_*j*_ ~ *N*(0, *τ*^2^);

*Time*_*tj*_ is the indicator of the pragmatic intervention in the *j*th school at time *t* and *θ* is the intervention effect (i.e., the change between pre- and post-intervention);

${S}_{j}^{(p)}$ is the *p*th covariate for the *j*th school (*p* = 1, …, *P*), the *P* covariates are the school characteristics including the indicator of the three school systems, and* γ*^(*p*)^ is the effect of ${S}_{j}^{(p)}$;

${C}_{ij}^{(q)}$ is the *q*th covariate for the *i*th student in the *j*th school (*q* = 1, …, *Q*), the *Q* covariates are the student characteristics, and* φ*^*q*)^ is the effect of ${C}_{ij}^{(q)}$; and.

*ε*_*tij*_ are random residuals such that *ε*_*tij*_ ~ *N*(0, *σ*^2^).

Because we will have a small sample of 30 school system administrators from the three school systems, we will conduct a mixed-methods descriptive study and integrate findings from qualitative interviews with these school system administrators as well as teachers, students, and staff with the results of the quantitative assessments on administrative climate and policy change from the MLM proposed above (Davis et al., 2019). By doing so, we will be able to assess administrator, teacher, student and staff experiences with the macro-level intervention as well as their perceptions of the impact of this intervention on policy change and school climate. These findings will also serve to inform next step broader implementation and dissemination of this intervention. The interviews will be conducted within 1 month following the yearlong macro-level Talking Circles and REIA intervention, using racially concordant, trained interviewers. Transcribed interviews will be analyzed using a content analysis technique that combines structural (e.g., intervention component, policy change barriers) magnitude coding (e.g., theme intensity) with inductive coding (e.g., variations in outcomes not captured by assessments) (Saldana, 2021). Findings from the interview data and assessment data will be compared in an integrative analysis (Tonkin-Crine et al., 2016), using mixed-method matrices for exploring convergence and dissonance between qualitative themes and assessment findings.

To analyze the data from phase II’s 1 year pretest–posttest factorial cRCT for evaluating the cross-level interventions at the meso- and micro-levels, we will conduct two-level MLM (individuals nested within schools), rather than three-level MLM, on our primary outcome, adolescent mental health, assessed at the posttest controlling for the pretest assessment. Such two-level MLM strategy will allow efficiently estimating and examining the cross-level intervention effects between the meso- and micro-levels which is the focus of this multilevel intervention study. The two-level MLM will also incorporate the estimated students’ perceptions of school system’s policy changes due to the macro-level intervention (MACRO) in phase I. The secondary outcomes among families and school personnel will be modeled in a similar manner. The two-level MLM for the primary outcome can be specified as follows (see S2 in the Supplementary Information for the two-level MLMs for the secondary outcomes):

2 $$\begin {aligned}{Y}_{ij}^{\left(1\right)}=\, &\mu +{\alpha }_{j}+{Y}_{ij}^{\left(0\right)}+{\beta }_{j}\times {{\text{MICRO}}}_{ij}\\&+{\theta }^{\left(1\right)}\times {{\text{MESO}}}_{j}+{\theta }^{\left(2\right)}\times {{\text{MICRO}}}_{ij}\\&+{\theta }^{\left(3\right)}\times {{\text{MESO}}}_{j}\times {{\text{MICRO}}}_{ij}\\&+\sum_{p=1}^{P}{\gamma }^{(p)}\times {S}_{j}^{(p)}\\&+{\varphi }^{(0)}\times {\mathrm{MACRO}}_{ij}\\&+\sum_{q=1}^{Q}{\varphi }^{(q)}\times {C}_{ij}^{(q)}+{\varepsilon }_{ij}\end {aligned}$$

where

${Y}_{ij}^{(1)}$ is the outcome assessed at the posttest for the *i*th student in the *j*th school (*i* = 1, … *n*_*j*_; *j* = 1, …, 30) and for this phase, *n*_*j*_ = 51 students;

${Y}_{ij}^{(0)}$ is the outcome assessed at the pretest for the *i*th student in the *j*th school;

*μ* is the mean of ${Y}_{ij}^{(1)}$ for the students in the schools receiving neither the meso- nor the micro-level intervention;

*α*_*j*_ is a random intercept for the *j*th school such that *α*_*j*_ ~ *N*(0, *τ*_*α*_^2^);

*β*_*j*_ is a random slope for the *j*th school such that *β*_*j*_ ~ *N*(0, *τ*_*β*_^2^);

*θ*^(*l*)^ (*l* = 1, 2, 3) are the main and interaction effects of the within- and cross-level interventions of *MESO*_*j*_ and *MICRO*_*ij*_;

${S}_{j}^{(p)}$ is the *p*th covariate for the *j*th school (*p* = 1, …, *P*), the *P* covariates are the school characteristics including the indicator of the three school systems, and* γ*^(*p*)^ is the effect of ${S}_{j}^{(p)}$;

${MACRO}_{ij}$ is the perceived school system’s policy change estimated from the macro-level intervention evaluated in Phase I for the *i*th student in the *j*th school, and *φ*^(0)^ is the effect of MACRO_*ij*_;

${C}_{ij}^{(q)}$ is the *q*th covariate for the *i*th student in the *j*th school (*q* = 1, …, *Q*), the *Q* covariates are the student characteristics, and *φ*^(*q*)^ is the effect of ${C}_{ij}^{(q)}$; and.

*ε*_*ij*_ are random residuals such that *ε*_*ij*_ ~ *N*(0, *σ*^2^).

It is worth noting that in this hypothetical study, we have 30 schools, and therefore have limited degrees of freedom for controlling school-level covariates. The specification of school-level covariates in the exemplary MLMs is for illustration only. In studies including more clusters, it would be appropriate to control for more school-level covariates. In addition, although we are not able to assess the interaction effect between macro- and meso-/micro-level interventions due to the limitation of this two-phase design, our MLM for phase II includes MACRO from phase I, which will allow us to control for the effects of the macro-level intervention. If the sample size permits, interactions between *MACRO* with *MESO* and/or *MICRO* would be of interest to estimate.

In this study, we are particularly interested in the estimates of phase I’s* θ* and phase II’s *θ*^(3)^ which will be used to answer our corresponding research aims. Additionally, the model includes a random slope which accounts for any variation in the effect of the micro-level intervention across schools. All the modeling and analyses can be conducted using commonly used statistical programs, such as SAS, SPSS, Stata, and R.

### Sample Size Consideration

There is no existing established single program that can be directly used to determine a sufficient sample size for this multiphase, hybrid pragmatic and factorial cRCT as a whole. Alternatively, we can consider the sample size phase by phase. Since the ICC of our primary outcome is unknown, we assume an ICC of 0.04, which is slightly more conservative than the midpoint of a documented ICC ranging between 0.01 and 0.07 for psychosocial adolescent health outcomes across schools (Shackleton et al., 2016).

For phase I that involves three-level MLM on a single group pretest–posttest design at level 1 without additional interventions at level 2 or level 3, we can simply use commonly used power analysis programs, such as PASS, 2023 (PASS, 2023; Power Analysis and Sample Size Software, n.d.), to first estimate a “design effect” sample size for detecting an expected effect size of the pretest–posttest pragmatic intervention effect at level 1 with an expected sufficient power. Then, we can calculate the required sample size for the phase I’s MLM analysis by using the “design effect” formula (Schoot et al., 2017, p. 5): *N*_Required_ = *N*_Design Effect_[1 + *I*CC(*K* − 1)], where *K* is the number of clusters, to adjust for the nesting effect of level 2 with level 3. For example, the results of power analysis using PASS (see S3 in Supplementary Information) revealed that the “design effect” sample size is 199 students/families that can detect a small effect size (standardized mean difference between pretest and posttest) of Cohen’s *d* = 0.20 for the pretest–posttest pragmatic intervention with a power of 0.80 at the significance level of 0.05. Then, the required number of students/families that are nested within 30 schools is 199 × [1 + 0.04 × (30 − 1)] = 430. In other words, 15 students/families from each of the 30 schools will be sufficient for phase I’s MLM analysis. In a similar calculation, with 10 school personnel from each of the 30 schools, the three-level MLM can detect a small effect size of Cohen’s *d* = 0.24 with a power of 0.80 at the significance level of 0.05 T.

To determine the sample size needed for the primary analysis in phase II, testing the cross-level interaction in our 2 × 2 factorial cRCT with randomization carried out at both the student/family and school levels, the R package *H2* × *2Factorial* (Tian et al., 2022) was used to compute the optimal sample size. For example, the results of power analysis using *H2* × *2Factorial* (see S4 in Supplementary Information) show that 3060 students/families (51 intervention students/families + 51 control students/families from each of the 15 intervention schools + 15 control schools) will be sufficient to detect a small effect size of Cohen’s *d* = 0.20 for the cross-level interaction effect of the multilevel intervention with a power of 0.80 at a significance level of 0.05. In the similar manner, 450 school personnel (15 from each of the 30 schools) will be able to detect a medium effect size of Cohen’s *d* = 0.53 for the cross-level interaction effect of the multilevel intervention with a power of 0.80 at a significance level of 0.05. While our study includes an equal number of families/students or school personnel within each school, this package can be used even with unequal cluster sizes.

It is worth noting that for simplicity, the power analysis described above did not consider controlling covariates which are shown in both Eqs. (1) and (2). Nevertheless, the estimated required sample sizes will provide sufficient power to detect small effects in most cases. That leaves room to control for a few key covariates if a medium effect size is anticipated. The selection of covariates should be informed by theoretical and empirical considerations.

## Discussion

Addressing systemic racism to improve mental health equity within schools requires rigorously tested multilevel interventions. However, there has been limited methodological guidance provided to researchers on how to rigorously test such multilevel interventions using a health equity lens that balances methodological rigor, practicality, and acceptability across stakeholder groups, especially within communities most affected by systemic racism. This paper provides an example of how to meticulously evaluate a theoretically based, multilevel intervention promoting mental health equity in a school system using an anti-racist approach. The methodological approach proposed can be adapted to other multilevel interventions that include strategies addressing macro-, meso-, and micro-levels of influence.

Multilevel interventions that intervene at the highest (i.e., macro and meso) levels are essential to disentangle the formal and informal policies and procedures that enable organizations to engage in discriminatory acts. These more distal level interventions (macro and meso) need to be combined with more proximal approaches to address the mental health consequences of these structures for racialized and minoritized communities. Since these policies and policy changes are likely to affect all individuals within an organization, the evaluation of multilevel interventions with macro- and meso-level components must be done with designs that allow for the allocation of groups to a treatment condition. Such designs must be complemented by analytic approaches that account for the nesting of individuals within groups.

Designing adequately powered cRCT studies is a complex undertaking. Cluster randomization is less efficient than individual randomization because outcome measures for individuals within the same cluster tend to be more similar than those of individuals of different clusters (van Breukelen & Candel, 2012). This reduction in efficiency results in cluster randomized trials requiring an increased sample size, which is a function of the cluster size as well as the intraclass correlation coefficient (ICC), a measure of the similarity in outcomes of individuals within a cluster. Therefore, the ICC must be estimated during the design process, though the ICC may be unknown or uncertain. In these situations, the most conservative approach is to assume the largest realistic ICC based on existing data. However, this can have significant financial implications, and may not be needed. An alternative approach, recommended by van Breukelen and Candel (2012), is to assume the ICC at the midpoint of an evidence-based ICC range. In our hypothetical examples, the known ICC range of psychosocial outcomes among high schools was documented in the literature as ranging between 0.01 and 0.07 (Shackleton et al., 2016) and we assumed an ICC (0.04) that was slightly more conservative than the midpoint ICC for our sample size calculation.

In order to have sufficient power to test the interaction between our meso-level and micro-level intervention, 30 high schools were needed. Significant time and resources will be required to implement such a large study, and substantial efforts will be needed to build relationships and trust with leaders in these school systems. It is often not feasible to work with such a large number of clusters, and having a limited number of clusters presents unique challenges. First of all, randomizing a few heterogeneous clusters increases the likelihood that potential sources of confounding are not distributed equally across study conditions (Murray et al., 2004; Turner et al., 2017a, b). Matching or stratifying on one or more characteristic prior to randomization can be used so that these characteristics are balanced across study conditions, leading to an increase in power and precision (Crespi, 2016). Some types of matching, however, may lead to other disadvantages, including complications of significance-testing of individual-level predictors, as described by Turner et al. (2017a). Thus, careful attention must be paid when designing cRCTs.

The methodological approach proposed to evaluate this hypothetical multilevel intervention has several limitations. Because there was no randomization at the school system level during phase I, our ability to make causal inferences on this macro-level intervention is hindered. For this reason, we complement the analysis of quantitative data with a qualitative evaluation to enhance our understanding of whether or how this intervention worked. Additionally, because all clusters (schools) received the macro-level intervention, interaction effects between the macro- and meso-level strategies and the macro- and micro-level strategies cannot be determined — but rather the additive effects examined. Finally, to evaluate the effect of the micro-level intervention on families, data will be collected from one parent or legal guardian in each family. While this approach allows for the family-level outcome to be modeled using the same two-level MLM approach as the student-level primary outcome, it may not fully capture the impacts of this intervention on all members of the family.

Despite the methodological limitations of the approach proposed, significant strengths are noted. First, phase I includes mixed methods that would allow for a robust and comprehensive understanding of how the restorative justice talking circles and the REIA impact decision makers (school administrators across the systems and schools) to influence policy change, and perceptions of teachers and students about potential changes. This can be used to inform the theory of change of these macro-level strategies and improve future efforts. The factorial cRCT of the meso- and micro level interventions will allow for a nuanced understanding of the potential synergy resulting from the presence of both levels of intervention on mental health outcomes in schools. This information will be critical to making evidence-based decisions on how to invest resources in schools to address systemic racism and improve the health and well-being of the school community, especially racial and ethnically minoritized students who are disproportionately affected by racist policies, climate, and behaviors in schools.

### Supplementary Information

Below is the link to the electronic supplementary material.

Supplementary file1 (DOCX 89 KB)
