# Correction: A Hybrid Pragmatic and Factorial Cluster Randomized Controlled Trial for an Anti-racist, Multilevel Intervention to Improve Mental Health Equity in High Schools

**DOI:** 10.1007/s11121-024-01674-x

**Published:** 2024-03-27

**Authors:** Marta I. Mulawa, Sharron L. Docherty, Donald E. Bailey, Rosa M. Gonzalez-Guarda, Isaac M. Lipkus, Schenita D. Randolph, Qing Yang, Wei Pan

**Affiliations:** 1 Duke University School of Nursing, Duke University, Durham, NC USA; 2 Duke Global Health Institute, Duke University, Durham, NC USA; 3 Department of Pediatrics, Duke University School of Medicine, Durham, NC USA; 4 Department of Population Health Sciences, Duke University School of Medicine, Durham, NC USA

**Correction to: Prevention Science** 10.1007/s11121-023-01626-x

The article “A Hybrid Pragmatic and Factorial Cluster Randomized Controlled Trial for an Anti racist, Multilevel Intervention to Improve Mental Health Equity in High Schools”, written by Mulawa, M.I., Docherty, S.L., Bailey, D.E. Jr., Gonzalez-Guarda, R.M., Lipkus, I.M., Randolph, S.D., Yang, Q., and Pan, W., was originally published electronically on the publisher’s internet portal on 04 January 2024 without open access. With the author(s)’ decision to opt for Open Choice the copyright of the article changed on 12 January 2024 to © The Author(s) 2024 and the article is forthwith distributed under a Creative Commons Attribution 4.0 International License, which permits use, sharing, adaptation, distribution and reproduction in any medium or format, as long as you give appropriate credit to the original author(s) and the source, provide a link to the Creative Commons licence, and indicate if changes were made. The images or other third party material in this article are included in the article’s Creative Commons licence, unless indicated otherwise in a credit line to the material. If material is not included in the article’s Creative Commons licence and your intended use is not permitted by statutory regulation or exceeds the permitted use, you will need to obtain permission directly from the copyright holder. To view a copy of this licence, visit http://creativecommons.org/licenses/by/4.0/.

The original article has been corrected.

