# Supplementary material for: A Hybrid Pragmatic and Factorial Cluster Randomized Controlled Trial for an Anti-racist, Multilevel Intervention to Improve Mental Health Equity in High Schools
Source: Prev Sci. 2024 Jan 4;25(Suppl 3):486–96. doi: 10.1007/s11121-023-01626-x (PMC11239747; doi:10.1007/s11121-023-01626-x)

**Supplementary Information for “*A Hybrid Pragmatic and Factorial Cluster Randomized Controlled Trial for an Anti-Racist, Multilevel Intervention to Improve Mental Health Equity in High Schools*”**

S1. Phase I Multilevel Modeling for Secondary Outcomes of Families and School Personnel

*For Families*

$Y_{tij}=\mu+\alpha_{j}+\theta\times{Time}_{tj}+\sum_{p=1}^{P} \gamma^{(p)}\times S_{j}^{(p)}+\sum_{q=1}^{Q} \varphi^{(q)}\times C_{ij}^{(q)}+\varepsilon_{tij}$ (1)

where

*Y_tij_* is the outcome (perceived school system’s policy change) for the *i*th family at time *t* in the *j*th school (*i* = 1, …, *n_i_*; *j* = 1, …, 30, *t* = 0, 1) and for this phase, *n_j_* = 15 families;

*μ* is the mean of *Y_tij_* in the pre-intervention condition at time 0;

*α_j_* is a random intercept for the *j*th school such that *α_j_* ~ *N*(0, *τ*^2^);

*Time_tj_* is the indicator of the pragmatic intervention in the *j*th school at time *t* and *θ* is the intervention effect (i.e., the change between pre- and post-intervention);

$S_{j}^{(p)}$ is the *p*th covariate for the *j*th school (*p* = 1, …, *P*), the *P* covariates are the school characteristics including the indicator of the three school systems, and *γ*^(^*^p^*^)^ is the effect of $S_{j}^{(p)}$;

$C_{ij}^{(q)}$ is the *q*th covariate for the *i*th family in the *j*th school (*q* = 1, …, *Q*), the *Q* covariates are the family characteristics, and *φ^q^*^)^ is the effect of $C_{ij}^{(q)}$; and

*ε_tij_* are random residuals such that *ε_tij_* ~ *N*(0, *σ*^2^).

*For School Personnel*

$Y_{tij}=\mu+\alpha_{j}+\theta\times{Time}_{tj}+\sum_{p=1}^{P} \gamma^{(p)}\times S_{j}^{(p)}+\sum_{q=1}^{Q} \varphi^{(q)}\times C_{ij}^{(q)}+\varepsilon_{tij}$ (2)

where

*Y_tij_* is the outcome (perceived school system’s policy change) for the *i*th school personnel at time *t* in the *j*th school (*i* = 1, …, *n_i_*; *j* = 1, …, 30, *t* = 0, 1) and for this phase, *n_j_* = 10 school personnel;

*μ* is the mean of *Y_tij_* in the pre-intervention condition at time 0;

*α_j_* is a random intercept for the *j*th school such that *α_j_* ~ *N*(0, *τ*^2^);

*Time_tj_* is the indicator of the pragmatic intervention in the *j*th school at time *t* and *θ* is the intervention effect (i.e., the change between pre- and post-intervention);

$S_{j}^{(p)}$ is the *p*th covariate for the *j*th school (*p* = 1, …, *P*), the *P* covariates are the school characteristics including the indicator of the three school systems, and *γ*^(^*^p^*^)^ is the effect of $S_{j}^{(p)}$;

$C_{ij}^{(q)}$ is the *q*th covariate for the *i*th school personnel in the *j*th school (*q* = 1, …, *Q*), the *Q* covariates are the school personnel characteristics, and *φ^q^*^)^ is the effect of $C_{ij}^{(q)}$; and

*ε_tij_* are random residuals such that *ε_tij_* ~ *N*(0, *σ*^2^).

S2. Phase II Multilevel Modeling for Secondary Outcomes of Families and School Personnel

*For Families*

$$Y_{ij}^{\left( 1 \right)}=\mu+\alpha_{j}+Y_{ij}^{\left( 0 \right)}+\beta_{j}\times{MICRO}_{ij}$$

$$+\theta^{\left( 1 \right)}\times{MESO}_{j}+\theta^{\left( 2 \right)}\times{MICRO}_{ij}+\theta^{\left( 3 \right)}\times{MESO}_{j}\times{MICRO}_{ij}$$

$+\sum_{p=1}^{P} \gamma^{(p)}\times S_{j}^{(p)}+\varphi^{(0)}\times{MACRO}_{ij}+\sum_{q=1}^{Q} \varphi^{(q)}\times C_{ij}^{(q)}+\varepsilon_{ij}$ (3)

where

$Y_{ij}^{(1)}$ is the outcome assessed at the posttest for the *i*th family in the *j*th school (*i* = 1, … *n_j_*; *j* = 1, …, 30) and for this phase, *n_j_* = 51 families;

$Y_{ij}^{(0)}$ is the outcome assessed at the pretest for the *i*th family in the *j*th school;

*μ* is the mean of $Y_{ij}^{(1)}$ for the family in the schools receiving neither the meso- nor the micro-level intervention;

*α_j_* is a random intercept for the *j*th school such that *α_j_* ~ *N*(0, *τ_α_*^2^);

*β_j_* is a random slope for the *j*th school such that *β_j_* ~ *N*(0, *τ_β_*^2^);

*θ*^(^*^l^*^)^ (*l* = 1, 2, 3) are the main and interaction effects of the within- and cross-level interventions of *MESO_j_* and *MICRO_ij_*;

$S_{j}^{(p)}$ is the *p*th covariate for the *j*th school (*p* = 1, …, *P*), the *P* covariates are the school characteristics including the indicator of the three school systems, and *γ*^(^*^p^*^)^ is the effect of $S_{j}^{(p)}$;

${MACRO}_{ij}$ is the perceived school system’s policy change estimated from the macro-level intervention evaluated in Phase I for the *i*th family in the *j*th school, and *φ*^(0)^ is the effect of *MACRO_ij_*;

$C_{ij}^{(q)}$ is the *q*th covariate for the *i*th family in the *j*th school (*q* = 1, …, *Q*), the *Q* covariates are the family characteristics, and *φ*^(^*^q^*^)^ is the effect of $C_{ij}^{(q)}$; and

*ε_ij_* are random residuals such that *ε_ij_* ~ *N*(0, *σ*^2^).

*For School Personnel*

$$Y_{ij}^{\left( 1 \right)}=\mu+\alpha_{j}+Y_{ij}^{\left( 0 \right)}+\beta_{j}\times{MICRO}_{ij}$$

$$+\theta^{\left( 1 \right)}\times{MESO}_{j}+\theta^{\left( 2 \right)}\times{MICRO}_{ij}+\theta^{\left( 3 \right)}\times{MESO}_{j}\times{MICRO}_{ij}$$

$+\sum_{p=1}^{P} \gamma^{(p)}\times S_{j}^{(p)}+\varphi^{(0)}\times{MACRO}_{ij}+\sum_{q=1}^{Q} \varphi^{(q)}\times C_{ij}^{(q)}+\varepsilon_{ij}$ (4)

where

$Y_{ij}^{(1)}$ is the outcome assessed at the posttest for the *i*th personnel in the *j*th school (*i* = 1, … *n_j_*; *j* = 1, …, 30) and for this phase, *n_j_* = 15 school personnel;

$Y_{ij}^{(0)}$ is the outcome assessed at the pretest for the *i*th school personnel in the *j*th school;

*μ* is the mean of $Y_{ij}^{(1)}$ for the school personnel in the schools receiving neither the meso- nor the micro-level intervention;

*α_j_* is a random intercept for the *j*th school such that *α_j_* ~ *N*(0, *τ_α_*^2^);

*β_j_* is a random slope for the *j*th school such that *β_j_* ~ *N*(0, *τ_β_*^2^);

*θ*^(^*^l^*^)^ (*l* = 1, 2, 3) are the main and interaction effects of the within- and cross-level interventions of *MESO_j_* and *MICRO_ij_*;

$S_{j}^{(p)}$ is the *p*th covariate for the *j*th school (*p* = 1, …, *P*), the *P* covariates are the school characteristics including the indicator of the three school systems, and *γ*^(^*^p^*^)^ is the effect of $S_{j}^{(p)}$;

${MACRO}_{ij}$ is the perceived school system’s policy change estimated from the macro-level intervention evaluated in Phase I for the *i*th school personnel in the *j*th school, and *φ*^(0)^ is the effect of *MACRO_ij_*;

$C_{ij}^{(q)}$ is the *q*th covariate for the *i*th school personnel in the *j*th school (*q* = 1, …, *Q*), the *Q* covariates are the school personnel characteristics, and *φ*^(^*^q^*^)^ is the effect of $C_{ij}^{(q)}$; and

*ε_ij_* are random residuals such that *ε_ij_* ~ *N*(0, *σ*^2^).

S3. The results of power analysis using PASS for Phase I’s Level-1 pretest-posttest pragmatic intervention effect before adjusting the nesting effect of Level 2 with Level 3.


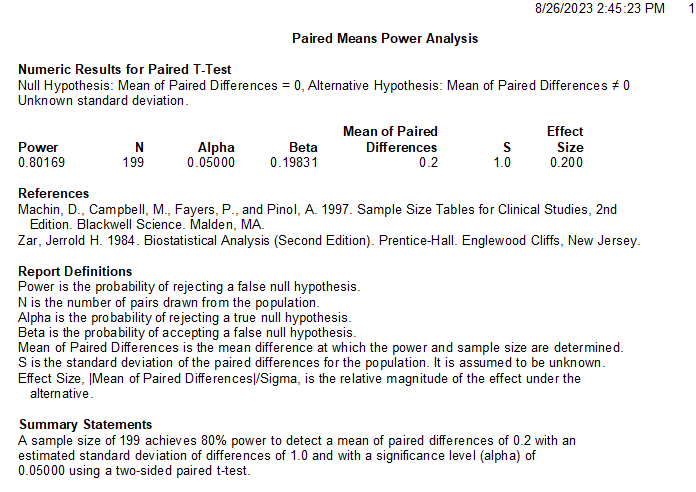


S4. The results of power analysis using *H2x2Factorial* for Phase II (*Note*: m_bar = the mean cluster size, delta_xz = the effect size of the cross-level interaction effect of the two interventions, rho = the intraclass correlation coefficient, estimand = the type of treatment effect estimand with "controlled" indicating that the main effects are controlled, test = the type of hypothesis test of interest with "interaction" indicating the cross-level interaction test for the two interventions).


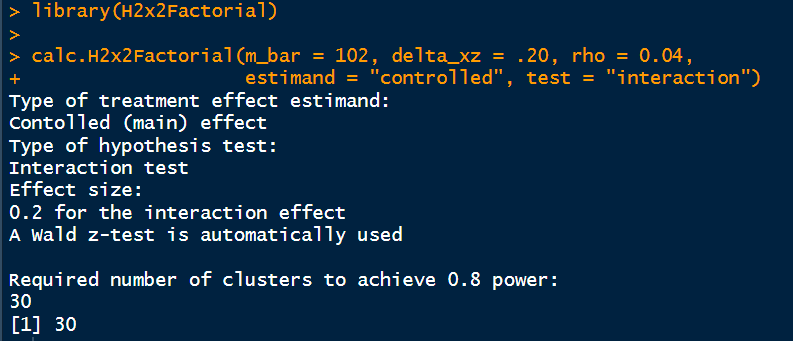

Supplement: Supplementary file 1 — Supplementary file1 (DOCX 89 KB) [file 11121_2023_1626_MOESM1_ESM.docx]
